# The Poor-Law Midwifery Schools

**Published:** 1908-01-25

**Authors:** 


### Nursing Administration.

### THE POOR-LAW MIDWIFERY SCHOOLS.

Among the many reasons for disquiet with regard to the working of the Midwives Act, and its approaching penal clauses, the inadequacy of the present midwifery training schools recognised by the Central Midwives Board, to prepare pupils for examination in sufficient number for the public requirements is not the least grave. These schools are, without exception, working at the highest point of efficiency, and are turning out every year as many trained midwives as their opportunities permit. Excluding the Poor-law schools, there are twelve institutions in London and twenty-one in the rest of England recognised as training schools for midwives by the Central Midwives Board, and in addition there are sixty-four recognised teachers. The number of candidates sent up by these recognised institutions and teachers in 1907 was 1,626. During the same period the number of recognised Poor-law schools was twenty, and of recognised teachers in Poor-law infirmaries twenty-three. The number of candidates sent up for examination from these sources was, however, only 139. It will not be contended by anyone acquainted with the needs of the country that this total of 1,765 candidates is satisfactory. A certain proportion, which at the last examination amounted to 16 per cent. were unsuccessful, thus still further reducing the number available for the public service. It is an urgent problem how the number can be increased. And in relation to this matter a serious difference of opinion has arisen between two important public bodies. It would certainly seem from the figures we have quoted that the Poor-law institutions do not contribute towards the spread of trained midwifery to the extent which the public have a right to expect. One hundred and thirty-nine midwives prepared for examination is a small number to show for the whole of England, including London. It is contended by many persons intimately acquainted with the workings of Poor-law institutions that this number would have been far higher had it not been for the discouraging attitude of the Central Midwives Board towards Poor-law institutions. It is argued that many infirmaries have been prevented from sending up excellent candidates for examination on account of the refusal of the Central Midwives Board to give them official recognition, and so great a hostility has been cultivated between the Local Government Board and the Central Midwives Board that it has been proposed in all seriousness to set up a dual authority, the Local Government Board laying down its own rules for "recognition," and bestowing the title of Midwifery Training School on its own institutions, without reference to the Central Midwives Board. We should regard such a measure as ill advised in every respect, and likely to have the worst possible effect in the public interests. When the facts are a little more clerely understood it will, we feel convinced, cease to be advocated by any impartial authorities.

We have before us a return showing the number of live births which took place during 1901-1905 in Union Workhouse Maternity Wards or Infirmaries serving populations of 100,000 and over, excluding London. We think it will surprise many people to learn that the total number of these births for the year 1905 was no more than 2,644, and this for the whole of England and Wales. Omitting certain institutions in which the number of births did not amount to twenty, we have a total of 2,561 births available for the purposes of instruction. Let it be supposed, and it is a daring supposition, that all of these births took place under conditions which may be held to constitute satisfactory training for pupil midwives, and we have a total of 133 midwives who might have qualified for examination during the year 1905. But out of this list seven of the more important institutions had already been recognised by the Central Midwives Board, and several others have since received recognition, so that the number of possible candidates lost is seen to be very small indeed. In point of fact, the Poor-law infirmaries have never been largely resorted to by the poor for the birth of their children. Even the unmarried mother, in destitution, deserted by her natural supporter, will submit to almost any deprivation in order to save her unborn child from the taint of being a "parish child." This fact must receive its proper weight in all deliberations on the problem of using Poor-law institutions as training grounds for midwives. It is not to the public interest that this strong sentiment of revulsion against parochial aid in childbirth shall be weakened. In London and in certain other large towns where Poor-law infirmaries are almost wholly dissevered from the associations of pauperism, this reluctance is greatly weakened; but it can hardly be contended that this indicates an improvement in general calibre on the part of those who accept this facile aid. As matters stand the number of births in by far the greater number of Unions is too small to allow of their training even one midwife during a year. Others show sufficient material for training one, two, rarely more than three. And it is clear that training conducted under these conditions is exposed to special dangers. A certain importance is attached at once to an infirmary which is branded officially as a midwifery training school, and to give this cachet to an institution which can at most receive three pupils for training during the year is to run some risk of bringing the term "Training School" into disrepute. The Central Midwives Board, alive to this danger, has suggested a compromise, which has been accepted with excellent results by many Poor-law institutions. This is to recognise as midwifery teacher one of the medical officers attached to the institution, thus enabling candidates to be presented for examination without more difficulty than in the more formally "recognised" infirmaries. This is the position of the Central Midwives Board with regard to candidates trained for their examinations in Poor-law institutions.

*(To be continued.)*

